# The Association Between Cardiorespiratory Fitness and Cognition Appears Neither Related to Current Physical Activity Nor Mediated by Brain-Derived Neurotrophic Factor in a Sample of Outpatients With Schizophrenia

**DOI:** 10.3389/fpsyt.2019.00785

**Published:** 2019-10-25

**Authors:** Tom Langerud Holmen, Jens Egeland, Eivind Andersen, Jon Mordal, Ole Andreas Andreassen, Thor Ueland, Therese Torgersen Bigseth, Gry Bang-Kittilsen, John Abel Engh

**Affiliations:** ^1^Division of Mental Health and Addiction, Vestfold Hospital Trust, Tønsberg, Norway; ^2^Department of Psychology, University of Oslo, Oslo, Norway; ^3^Faculty of Humanities, Sports and Educational Science, University College of Southeast Norway, Notodden, Norway; ^4^NORMENT Centre, Division of Mental Health and Addiction, Oslo University Hospital, Oslo, Norway; ^5^Institute of Clinical Medicine, University of Oslo, Oslo, Norway; ^6^Research Institute of Internal Medicine, Oslo University Hospital, Rikshospitalet, Oslo, Norway; ^7^KG Jebsen Thrombosis Research and Expertise Centre, University of Tromsø, Tromsø, Norway

**Keywords:** psychosis, schizophrenia, cognition, physical, activity, exercise, CRF, BDNF

## Abstract

**Objective:** We investigated whether levels of current physical activity (PA) contribute to the established relationship between cardiorespiratory fitness (CRF) and cognition in schizophrenia and whether brain-derived neurotrophic factor (BDNF) or its precursor proBDNF mediates this relationship.

**Method:** Sixty-one outpatients with schizophrenia spectrum disorders participated. Neurocognition was assessed with the Wechsler Adult Intelligence Scale (WAIS) and nine subtests from the MATRICS battery comprising a neurocognitive composite score (NCS). CRF was assessed with peak oxygen uptake (VO_2peak_) measured directly during a maximum exercise test. Current PA levels were objectively assessed by an accelerometer worn for four consecutive days. BDNF and proBDNF were measured in fasting blood. Four serial parallel mediation analyses and two additional parallel mediation analyses were conducted, while controlling for age and sex at all levels.

**Results:** No direct effects were found between PA measures and WAIS or NCS. No significant mediating effects of CRF or BDNF/proBDNF were detected.

**Conclusion:** The results do not support the hypothesis that PA contributes to the naturally occurring relationship between CRF and cognition in schizophrenia or the hypothesis that BDNF or proBDNF mediates this relationship. The results arguably support the assumption that the association between CRF and cognition in schizophrenia is established developmentally early.

**Clinical Trial Registration:** www.ClinicalTrials.gov, identifier NCT02205684.

## Introduction

Individuals with schizophrenia display profoundly low levels of cardiorespiratory fitness (CRF) (1–4) and impaired cognition is a clinical feature in schizophrenia (5, 6). Thus, the relationship between CRF and cognition is of special interest with regard to this patient group. In a previous study, we found that CRF explained 8.2% and 9.0% of the variance in general intellectual ability (IQ) and state-sensitive cognitive functions (processing speed, attention, working memory, learning and executive functions), respectively (6, 7). The association between CRF and cognition appeared significant beyond the impact of negative psychotic symptom load and other potential confounders. Moreover, the association was explained by a modality-specific relationship between CRF and a verbal cognitive factor (5, 7).

Moderate to vigorous physical activity (MVPA), as in exercise, is a central CRF-enhancing lifestyle factor (8). A meta-analysis of 10 exercise intervention studies with individuals with schizophrenia reported significant effects of increased CRF on overall cognitive performance, as well as for specific domains such as working memory, attention/vigilance, and social cognition (9). Hence, higher levels of MVPA may improve cognition through increased CRF. Conversely, better cognitive abilities may provide better opportunities for a healthy lifestyle, including higher levels of MVPA and thus improved CRF, and these factors may interact in a mutually reinforcing manner (10). Presumably, such effects would be reflected in current naturally occurring associations between physical activity (PA) and cognition, in which CRF would appear as a mediator. One study has shown positive associations between moderate PA and measures of processing speed and verbal working memory among 30 older individuals with schizophrenia (aged >55) (11). Another study showed associations between light PA and tasks measuring attention/concentration and speed of processing in a sample of 199 individuals with schizophrenia (12). However, none of these studies included measures of CRF or other potential mediators to these relationships. In a previous study, we found that only a more general and less vigorous indicator of current PA, number of steps per day, was significantly associated with CRF in schizophrenia (13). This indicates that variation in CRF among individuals with schizophrenia, being less fit than the population at large, should be studied with measures sensitive to less intensive PA—such as walking (steps per day). There may also be routes of influence between PA and cognition which do not involve CRF. For example, the bioenergetic effects hypothesis centers on the mediating role of brain-derived neurotrophic factor (BDNF) (14).

BDNF plays a modulating role in dopamine, serotonin, and GABA systems (15) and is associated with neuronal development and plasticity through synaptic long-term potentiation (16, 17) and, thus, with learning and memory. BDNF may traverse the blood-brain barrier, indicating that systemic levels may reflect brain levels. Individuals with schizophrenia are characterized by low circulating levels of BDNF, and there is a positive association between levels of BDNF and reasoning/problem solving in schizophrenia (18). BDNF in its precursory form, proBDNF, may have effects opposite from its mature version (19), which bespeaks the complexity of its functions. One study has shown that cognitive remediation leads to an increase in BDNF in individuals with schizophrenia (20); however, this was not replicated in a slightly larger sample (21). Moreover, Kimhy and colleagues found that aerobic exercise improved levels of BDNF in a sample of individuals with schizophrenia, as compared to controls (22). Aerobic exercise and other forms of PA may influence the blood-brain barrier permeability and the release of growth factors, including BDNF, in central and peripheral tissue. PA may also contest metabolism-related, proinflammatory processes which obstruct the production and functioning of growth factors, including BDNF (23). Consequently, BDNF may act as a mediator between PA and cognition. Such an effect may be related directly to acute bouts of PA, such as exercise, but also to long-term low level PA sustained over time (23).

**Figure 1:**
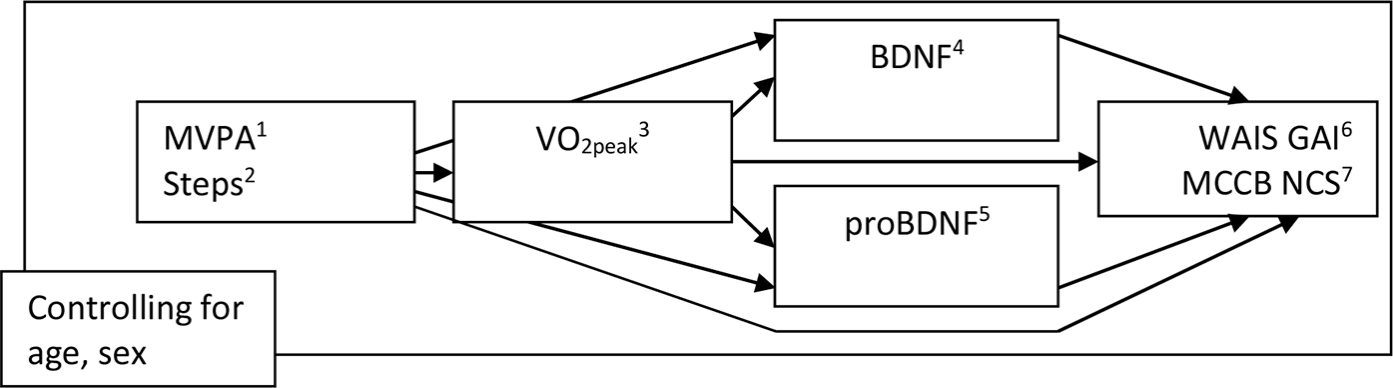
The effect of MVPA and Steps on WAIS GAI and MCCB NCS, directly and through VO2peak and/or BDNF and proBDNF (n = 61): Visualization of pathways. ^1^Moderate and vigorous physical activity, minutes per day. ^2^Number of steps per day. ^3^VO2peak: ml/kg/min. ^4^Brain-derived neurotrophic factor. ^5^Pro-brain-derived neurotrophic factor. ^6^Wechsler Adult Intelligence Scale version IV General. Ability Index. ^7^MATRICS Consensus Cognitive Battery Neurocognitive. Composite Score.

CRF as measured by maximal oxygen uptake (VO_2peak_) explains some of the variance in cognition in schizophrenia (6). It is not clear what causes this relationship or what mediates it. In the present study, we investigated the potential impact of PA on cognition through the potentially mediating levels of CRF and BDNF/proBDNF. In previous studies, we have shown that CRF is associated with cognition in schizophrenia and also that a general measure of PA in steps per day—but not the more intensive measure of MVPA—is associated with CRF (6, 13). However, whether the effect of steps extends from CRF to cognition, and whether steps or MVPA influences cognition directly or through the second mediating level of BDNF, has not been assessed. The pathways are visualized in **Figure 1**.

We hypothesize that current levels of PA (steps per day and MVPA) will be positively associated with cognition in this sample of individuals with schizophrenia, with CRF and/or BDNF mediating the relationship between PA and cognition. To explore the potential role of BDNFs precursor in this model, we will include proBDNF as an alternative mediator. We will analyze these relationships through four mediation analyses; with steps and MVPA as alternating independent variables and two different composite measures of cognition as alternating dependent variables; with CRF, BDNF, and proBDNF as serial and parallel mediators respectively; while controlling for age and sex at all levels in the models.

## Materials and Methods

### Design

The study was conducted on baseline data from the randomized, controlled, observer-blinded clinical trial “Effects of Physical Activity in Psychosis” (EPHAPS) [ClinicalTrials.gov, registration number NCT02205684 (24)]. The study was approved by the Regional Committee for Medical and Health Research Ethics of Southern and Eastern Norway (file number 2014/372/REK SØR-ØST).

### Participants

Sixty-one participants aged 20–64 years were recruited from August 2014 through February 2017 from catchment area-based and publicly funded outpatient psychiatric clinics in Vestfold County, Norway. A subgroup of patients was referred from primary health care to the outpatient clinics for participation in the project specifically. Eligible for the study were patients who fulfilled the Diagnostic and Statistical Manual of Mental Disorders (5th ed.; DSM-V; 25) criteria for schizophrenia spectrum disorder (schizophrenia, schizoaffective disorder, and schizophreniform disorder). Demographic and clinical characteristics are presented in **Table 1**. Diagnosis was established using the Structured Clinical Interview for Diagnostic and Statistical Manual of Mental Disorders axis I [SCID I (26)]. Interviews were conducted by a clinical psychologist or a specialist in psychiatry. Members of the assessment staff attended a course based on the SCID training program at the University of California Los Angeles (27) and all participated in diagnostic consensus meetings. Additional inclusion criteria were age between 18 and 67 and understanding and speaking a Scandinavian language. Exclusion criteria were pregnancy, chest pain during CRF test, unstable angina pectoris, recent myocardial infarction, uncontrollable cardiac arrhythmia, severe hypertension (>180/110 mmHg), comorbid diagnosis of mild mental retardation, or other medical conditions incompatible with participation. Initial information about the study was given to eligible patients by clinical staff in the outpatient clinic or in primary health services. Contingent on understanding the nature of the research and willingness to participate, written consent was obtained by a project coworker.

**Table 1:** Demographic and clinical characteristics of participants (n = 61).

Attribute	Mean (SD)	
Male *n* = 34 (56%)		
Age (years)	36.4	(13.3)
Education (years)	12.2	(2.4)
Duration of illness (years)	14.7	(11.7)
GAF-S^1^*	43.6	(7.7)
GAF-F^2^*	44.4	(7.9)
PANSS^3^ total	64.2	(17.3)
WAIS-IV GAI^4^	88.0	(16.2)
MCCB NCS^5^	35.0	(8.3)
VO_2peak_ (ml·kg^–1^·min^–1^)	29.9	(11.8)
MVPA^6^	26.7	(24.2)
Steps^7^	5235.3	(2930.1)
BDNF^8^	1.0	(0.9)
proBDNF^9^	0.7	(0.3)
Antipsychotics DDD^10^ **	1.7	(0.9)

^1^Global Assessment of Functioning-Symptom scale; ^2^ Global Assessment of Functioning-Function scale; ^3^Positive and Negative Syndrome Scale;

^4^Wechsler Adult Intelligence Scale version IV General Ability Index.

^5^MATRICS Consensus Cognitive Battery Neurocognitive

Composite Score.

^6^Moderate and vigorous physical activity, minutes per day;

^7^Number of steps per day; ^8^Brain-derived neurotrophic factor;

^9^Pro-brain-derived neurotrophic factor. ^10^Defined Daily Doses.

*Data on GAF-scores were missing for one individual.

**Sixty individuals received antipsychotic medication.

Sixty participants received antipsychotic medical treatment. Defined daily doses (DDD) were calculated in accordance with guidelines from the World Health Organization Collaborating Center for Drug Statistics Methodology (http://www.whocc.no/atcdd). The DDD is the assumed average maintenance dose per day for a drug used for its main indication on adults, providing a fixed unit of measurement independent of dosage form and thus a rough estimate of pharmaceutical drugs consumption. Furthermore, 13 participants received antidepressant medication, nine of which contained selective serotonin reuptake inhibitors.

### Procedure

The current study was based on neurocognitive and clinical assessments, a CRF test, a blood test from which BDNF and proBDNF were extracted, and accelerometer data. All assessments were conducted within a two-week period. Neurocognition was assessed with the six subtests of the Wechsler Adult Intelligence Scale version 4 (2008, NCS Pearson Inc. San Antonio, TX, USA), which comprise the General Ability Index (WAIS-IV GAI) and may be regarded as a core IQ measure not contaminated by state-dependent reductions in processing speed and memory. Furthermore, nine of the 10 subtests in the MATRICS consensus cognitive battery were applied as measures of the state-sensitive cognitive functions, yielding a neurocognitive composite score (MCCB NCS). The MCCB NCS assesses cognitive functioning in six domains: speed of processing, attention/vigilance, working memory, verbal learning, visual learning, and problem solving (28). The Norwegian version of MCCB has retained the original psychometric properties (29) with the possible exception of the test intended to measure social cognition (the Mayer-Salovey-Caruso Emotional Intelligence Test), which was not included in the current study.

Peak oxygen uptake (VO_2peak_) was measured during a maximum exercise test on a treadmill using a modified Balke protocol (30), where speed was held constant at 5 km/h and the inclination angle was increased by one degree every minute until exhaustion. Gas exchange was sampled continuously into a mixing chamber every 30 s by having the participants breathe into a Hans Rudolph two-way breathing valve (2,700 series, Hans Rudolph Inc., Kansas City, MO, USA) connected to a Jaeger Oxycon Pro gas analyzer (Erich Jaeger GmbH, Hoechberg, Germany), which measures the oxygen and carbon dioxide content. Before conducting the CRF test, somatic health was thoroughly assessed through general medical examination, including medical history, medication record, and physical examination with blood pressure assessment and electrocardiography. For various reasons, some individuals may fail to reach a true VO_2max_, and for the sake of conservative reporting, we therefore use the term VO_2peak_.

PA was assessed using the GT3X+ accelerometer from Actigraph (ActiGraph, LLC, Pensacola, FL, USA). Participants were instructed to wear the accelerometer over the left hip while awake for four consecutive days. In order to ensure an even distribution between the two-week phases, the participants were instructed to use the accelerometer for two weekdays and the two weekend days. The accelerometer is a seismic instrument which continuously measures acceleration, and raw data from this instrument are called “counts,” which are the sum of accelerations in a given time period (4). Analyses were restricted to participants who wore the accelerometer for a minimum of 10 h per day for two days or more. MVPA is defined as the number of minutes spent in all activity ≥ 2020 counts per minute and calculated from the number of minutes spent at this activity level per day. “Steps” is the registered number of steps per day. Participants who wore the monitor for two days or more were included in the analysis (*n* = 61). 9 participants wore the accelerometer for 2 days, 13 wore it for 3 days, 37 wore it for 4 days, and 2 wore it for 5 days.

Fasting blood samples were obtained from all participants during morning hours within the two-week assessment period. Samples were processed to platelet poor plasma and frozen at −80°C within 2 h. Enzyme immunoassay development kits from R&D Systems (Stillwater, MN) were used to measure proBDNF (DY3175) and BDNF (DY248). Intraassay and interassay variation was <10%.

### Statistical Analysis

Analyses of skewness and kurtosis indicated that data were normally distributed for the two measures of cognition (WAIS GAI and MCCB NCS) and CRF. One outlier was identified for both MVPA and steps and recoded into the second highest value in the data set. Two outliers were identified for proBDNF and recoded into the third highest value in the data set. The variables of MVPA, steps, and BDNF were log-transformed.

Statistical analyses were made using The Statistical Package for the Social Sciences (IBM SPSS Version 22.0, IBM Corp, Armonk, NY, USA), with the addition of the PROCESS program for mediation/moderation analyses (31). Four serial parallel mediation analyses and two additional parallel mediation analyses were conducted with PROCESS, using ordinary least squares path analysis. Significance levels were set at 5% for all analyses of direct effects between independent and dependent variables. 95% bootstrap confidence intervals with iteration set to 10,000 were calculated for all indirect effects involving mediators [unstandardized effects, as recommended by Hayes (31)]. The four main analyses included MVPA and steps as alternating independent variables and WAIS GAI and MCCB NCS as alternating dependent variables, and with VO_2peak_, BDNF, and proBDNF as serial and parallel mediators respectively, while controlling for age and sex at all levels in the models. Pathways are visualized in **Figure 1**.

Accelerometer data were obtained for 61 participants, whereas BDNF and proBDNF data were available for 78 participants. In order to maximize utilization of data, two additional analyses were done with sole focus on the mediating effects of BDNF and proBDNF as parallel mediators in this slightly larger sample. VO_2peak_ was entered as independent variable and WAIS GAI and MCCB NCS as alternating dependent variables, while controlling for age and sex at all levels in the models.

## Results

Four serial parallel mediation analyses using ordinary least squares path analysis were conducted (*n* = 61), and no significant direct effects were found between MVPA or steps on one side and WAIS GAI or MCCB NCS on the other (MVPA–WAIS GAI: *c’* = −4.47, *p* = 0.37; MVPA–MCCB NCS: *c’* = −3.27, *p* = 0.22; steps–WAIS GAI: *c’* = −10.04, *p* = 0.24; steps–MCCB NCS: *c’* = −2.26, *p* = 0.63). Bootstrap confidence intervals based on 10,000 bootstrap samples for all indirect effects in all analyses all included zero, prohibiting rejection of respective null hypotheses, thus leaving any mediation effects from VO_2peak_ and/or BDNF and/or proBDNF nonsignificant. Two additional parallel mediation analyses (*n* = 78) showed direct effects between VO_2peak_ and both WAIS GAI (*c’* = 0.58, *p* = 0.01) and MCCB NCS (*c’* = 0.31, *p* = 0.01), as expected from previous studies (6, 7). However, results from the main analyses were retained; no significant mediating effects from BDNF or proBDNF were found in this slightly increased sample. All indirect effects, with respective bootstrap confidence intervals, are presented in **Table 2**.

**Table 2:** Mediation effects (n = 61).

Path	Mediation effect	95% bootstrap CI (LL, UL)
MVPA^1^-VO_2peak_^2^-WAIS GAI^3^	0.64	–2.05, 4,16
MVPA-BDNF^4^-WAIS GAI	–0.21	–2.06, 1.45
MVPA-proBDNF^5^-WAIS GAI	–0.05	–1.91, 2.00
MVPA-VO2_peak_-BDNF-WAIS GAI	–0.03	–0.59, 0.32
MVPA-VO2_peak_-proBDNF-WAIS GAI	0.05	–0.31, 0.66
MVPA-VO2_peak_-MCCB NCS^6^	0.37	–1.21, 2.39
MVPA-BDNF-MCCB NCS	0.10	–0.83, 1.25
MVPA-proBDNF-MCCB NCS	–0.02	–0.85, 0.89
MVPA-VO2_peak_-BDNF-MCCB NCS	0.02	–0.26, 0.26
MVPA-VO2_peak_-proBDNF-MCCB NCS	0.02	–0.15, 0.25
Steps^7^-VO2_peak_-WAIS GAI	3.88	–1.69, 12.71
Steps -BDNF-WAIS GAI	0.01	–2.28, 2.12
Steps-proBDNF-WAIS GAI	0.16	–2.74, 5.05
Steps-VO2_peak_-BDNF-WAIS GAI	–0.21	–1.86, 0.79
Steps-VO2_peak_-proBDNF-WAIS GAI	0.23	–0.73, 1,92
Steps-VO2_peak_-MCCB NCS	1.97	–1.85, 6.37
Steps-BDNF-MCCB NCS	0.00	–1.42, 1.25
Steps-proBDNF-MCCB NCS	0.07	–1.38, 1.91
Steps-VO2_peak_-BDNF-MCCB NCS	0.05	–0.74, 0.84
Steps-VO2_peak_-proBDNF-MCCB NCS	0.10	–0.38, 0.73
VO2_peak_-BDNF-WAIS GAI*	–0.03	–0.15, 0.04
VO2_peak_-proBDNF-WAIS GAI*	0.00	–0.05, 0.08
VO2_peak_-BDNF-MCCB NCS*	–0.01	–0.07, 0.03
VO2_peak_-proBDNF-MCCB NCS*	0.00	–0.03, 0.03

^1^Moderate and vigorous physical activity, minutes per day; ^2^VO2_peak_: ml/kg/min; ^3^Wechsler Adult Intelligence Scale version IV General Ability Index.

^4^Brain-derived neurotrophic factor; ^5^Pro-brain-derived neurotrophic factor; ^6^MATRICS Consensus Cognitive Battery Neurocognitive Composite Score.

^7^Number of steps per day; *Additional analyses with BDNF and proBDNF as sole mediators (n = 78).

## Discussion

In the current study, neither MVPA nor steps appeared to exert significant effects on cognition either directly or indirectly through any of the mediators. Moreover, neither BDNF nor proBDNF appeared to mediate the relationship between CRF and cognition.

Five hypotheses have been proposed on specific mediator mechanisms between PA and cognition (14). The cardiovascular fitness hypothesis holds that improved oxygen transport and metabolism lead to improved neurotransmitter function (32), and the cerebrovascular reserve hypothesis points to the ability of cerebral blood vessels to respond to increased metabolic demand and chemical, mechanical, or neural stimuli (33). The motor fitness hypothesis focuses on differential ripple effects from increased movement- and coordination-specific perceptual and cognitive demand (14). In a similar vein, the selective improvements hypothesis suggests that executive functions have the greatest potential to respond to aerobic exercise, as these are disproportionately affected by ageing (34). Finally, and partly in response to phenomena unexplained by these theories such as rapid improvements in cognition shortly after physical exercise (35), the bioenergetic effects hypothesis centers on the mediating role of BDNF. It focuses on this growth factor’s ability to enhance cellular energy metabolism and reduce oxidative stress; animal studies on blocking BDNF-receptors in the hippocampus indicate that physical fitness alone does not improve cognition (36).

The current results do not lend support to the bioenergetic effects hypothesis. None of the two measures of BDNF appeared to have any significant mediating effect between PA/CRF and cognition. Furthermore, in a previous study (5), we did not find support for the effects proposed by the motor fitness or the selective improvements hypotheses to be present in this sample of individuals with schizophrenia. There was no evidence of a proximal link between CRF and cognitive functions believed to be more involved during exercise, or a selective effect on executive functions. Contrarily, we found that CRF was selectively associated with a verbal cognitive factor. However, it must be noted that the proposed effect of the motor fitness hypothesis is not limited to CRF-enhancing activities but includes also less metabolically demanding activities such as coordination tasks. Any such non-CRF-dependent effects would not have been detected in this previous study. Moreover, we have previously established that number of daily steps is associated with CRF (13), and that CRF is associated with cognition (6, 7). Still, the current results show that the effect does not carry over from steps, *via* CRF, to cognition. This indicates independent, partial effects between steps and CRF and between CRF and cognition respectively, and/or a signal not strong enough to bear through the mediating level of CRF. Thus, the results appear equivocal as to the presence of effects proposed by the cerebrovascular reserves and cardiovascular fitness hypotheses. Also, the results do not support a complementary sixth hypothesis of a converse or reciprocal effect between cognition and PA- that individuals with higher cognitive functioning become more physically active.

There may be alternative reasons why we do not find support for the hypothesized effects. Importantly, the first five hypotheses described above primarily address mechanisms behind reported effects of various exercise interventions, which may not be present in the naturally occurring, long-term developing relationships between the same variables. Such relationships may be limited to a subsample of participants who regularly engage in exercise, and otherwise overshadowed by other factors, such as common genetic factors behind CRF, cognition, and/or schizophrenia. For example, BDNF is associated with CRF, cognition, and schizophrenia (18, 23, 37), and a mediating role between CRF and cognition may be obscured by disorder-specific effects in schizophrenia.

The study has some limitations. Although the objective assessment of PA provides validity (38), the limited number of days the participants wore the accelerometer (two to five) may have compromised the representativeness of the measure. Diurnal and daily variations in BDNF, for a variety of reasons, may have occluded its potentially mediating effect between PA/CRF and cognition. This includes the possible impacts of menstrual phase for the female participants and selective serotonin reuptake inhibitors for the nine participants receiving medication containing this active substance. In this study, we have sought to further explain an established relationship between the relatively stable attributes of CRF and cognition, by considering the roles of two presumably less stable factors of PA and BDNF. The potential variability of these may have obstructed the detection of genuine effects. Moreover, in the absence of a control group, we do not know whether the results are specific for individuals with schizophrenia or also apply to the general population.

In previous studies, we have found that the naturally occurring association between CRF and cognition in schizophrenia is tied to a verbal cognitive factor, on which the Verbal Comprehension Index of WAIS IV loads highly (7). We have found no indications of a stronger link between CRF and cognitive functions thought to be more state-sensitive or modifiable through life-style factors (6). Furthermore, we found that the relationship between CRF and cognition could not be explained by current lifestyle related factors such as metabolism-based illnesses or smoking. In the current study, we detected no significant influence from present levels of PA on the association between CRF and cognition. Also, no mediating effects of BDNF or its precursor proBDNF were found in the relationships between PA/CRF and cognition, in this sample. Lastly, there was no indication that the association between CRF and cognition can be explained by better cognitively functioning individuals being more active.

## Data Availability Statement

The datasets generated for this study are available on request to the corresponding author.

## Ethics Statement

The studies involving human participants were reviewed and approved by The Regional Committee for Medical and Health Research Ethics of Southern and Eastern Norway (file number 2014/372/REK SØR-ØST). The patients/participants provided their written informed consent to participate in this study.

## Author Contributions

All authors contributed to the manuscript in accordance with the Vancouver Protocol.

## Funding

This work was supported by Vestfold Hospital Trust, Division of Mental Illness and Drug Addiction and Vestfold Hospital Trust, Research and Development (grant number 2013/842), Norwegian Extra Foundation for Health and Rehabilitation through EXTRA funds (grant number 2013/2/0183), Norwegian Research network in Severe Mental Illness (NORSMI; grant number 39383/2011-13) and Torgeir Lindvig’s Trust (grant number 2013).

## Conflict of Interest

The authors declare that the research was conducted in the absence of any commercial or financial relationships that could be construed as a potential conflict of interest.
